# The complete chloroplast genome of *Echinochloa haploclada*

**DOI:** 10.1080/23802359.2021.1982654

**Published:** 2021-09-30

**Authors:** Bowen Jiang, Sangting Lao, Dongya Wu, Longjiang Fan, Chu-Yu Ye

**Affiliations:** Institute of Crop Sciences, College of Agriculture and Biotechnology, Zhejiang University, Hangzhou, China

**Keywords:** Chloroplast genome, *Echinochloa haploclada*, *Echinochloa crus-galli*, weed

## Abstract

The genus *Echinochloa* (Poaceae) includes orphan crops and important agricultural weeds. Here, we assembled the complete chloroplast genome of a diploid *Echinochloa* species (*E. haploclada*). The chloroplast genome is 139,844 bp in length, which includes a large single copy region (81,893 bp), a small single copy region (12,533 bp) and two separated inverted repeat regions (45,418 bp). A total of 119 unique genes were annotated, consisting of 83 protein-coding genes, 32 tRNA genes and 4 rRNA genes. Hexaploid *E. crus-galli*, one of the most serious weeds worldwide, was derived from a hybrid between tetraploid *E. oryzicola* and an unknown diploid species. Based on chloroplast genomes of eight *Echinochloa* species (varieties), the phylogenetic analysis showed that *E. crus-galli* clustered firstly with diploid *E. haploclada* rather than tetraploid *E. oryzicola*, supporting previous assumption that *E. oryzicola* is the paternal donor of *E. crus-galli*.

The genus *Echinochloa* (Poaceae) includes orphan crops (barnyard millet) and many problematic weeds in agricultural fields, e.g. hexaploid *E. crus-galli* (the dominant *Echinochloa* species) is one of the most detrimental weeds in rice paddies (Ye and Fan 2021). Chloroplast genome sequences are useful for understanding plant origin and evolution. So far, there are at least eight *Echinochloa* species (varieties) with released chloroplast genomes (NCBI Organelle Genome Resources), e.g. hexaploid *E. crus-galli*, tetraploid *E. oryzicola* and hexaploid *E. colona* (Ye et al. 2014; Nah et al. 2016; Perumal et al. 2016; Lee et al. 2017; Piot et al. 2018). Among them, however, the diploid species are still lacking, which hinders our understanding of the evolution of *Echinochloa* species.

In this study, we assembled the complete chloroplast genome of a diploid *Echinochloa* species, *E. haploclada,* which was collected in Kenya, near Muhaka (04°20.201 S, 39°28.137 E) and deposited in the Herbarium of Zhejiang University (accession number HZU60206921), based on whole-genome high-throughput sequencing data generated by us previously (Ye et al. 2020). After quality control with NGSQCToolkit v2.3 (Patel and Jain 2012), the clean data was applied in *de novo* assembly by NOVOPlasty v3.6 (Dierckxsens et al. 2017) using the *Panicum virgatum* (neighboring genus of *Echinochloa*) complete chloroplast genome (GenBank accession number NC_015990) as a reference. Genome annotation was performed by the GeSeq online (Tillich et al. 2017). The assembled genome sequences and annotation information have been submitted in National Genomics Data Center (NGDC, China) under accession number GWHBAUW01000000 and Genbank under accession number MW672445.1.

The total length of *E. haploclada* chloroplast genome is 139,844 bp. Similar to most angiosperm chloroplast genomes, this genome exhibited a distinct quadripartite structure, including a pair of inverted repeats (IRa and IRb, 22,709 bp each), the large single-copy region (LSC, 81,893 bp) and the small single-copy region (SSC, 12,533 bp). The GC contents of the IR, LSC and SSC regions are 36.4%, 33.1%, and 44.0%, respectively. A total of 119 unique genes were annotated and 24 genes, including 10 protein-coding genes, 8 tRNA genes and 4 rRNA genes, were duplicated in the IR regions. Among these 119 genes, there are 83 protein-coding genes, 32 tRNA genes and 4 rRNA genes, and 7 genes contained introns (6 and 1 genes contained 1 and 2 introns, respectively).

To investigate the evolutionary position of *E. haploclada* among *Echinochloa* species, we built a phylogenetic tree of eight *Echinochloa* species (varieties) and four other sister groups (*Alloteropsis*, *Panicum*, *Setaria* and *Digitaria*) based on complete chloroplast genome sequences using *Oryza sativa* as an outgroup. We first performed alignment by MAFFT v7.310 (Katoh et al. 2002) with the parameter ‘auto’ (‘FFT-NS-2’ was finally assigned by MAFFT). Then, IQ-tree v1.6.12, an effective algorithm for estimating maximum-likelihood phylogenies, was used to construct a phylogenetic tree with recommended setting ‘-m MFP -bb 1000 -bnni’ (GTR + F+R4 model was finally selected) (Nguyen et al. 2015). Finally, the tree was illustrated and modified using iTOL (Letunic and Bork 2019).

The phylogeny showed that *E. haploclada* first clustered with *E. crus-galli* forming a monoclade (Figure 1). Compared to tetraploid *E. oryzicola*, diploid *E. haploclada* showed closer relationship to hexaploid *E. crus-galli*. The dominant hexaploid species *E. crus-galli* was arisen from the hybridization between tetraploid *E. oryzicola* and an unknown diploid species. It has been assumed that *E. oryzicola* is the paternal donor based on nuclear DNA internal transcribed spacer and chloroplast DNA segments (Aoki and Yamaguchi 2008). Additionally, our previous study revealed that *E. haploclada* is not but is very close to the direct ancestor of *E. crus-galli* based on nuclear genome sequences (Ye et al. 2020). Therefore, considering the maternal inheritance of chloroplast genome, the phylogenetic analysis in this study supported that tetraploid *E. oryzicola* is the paternal donor while an unknown diploid species (close to *E. haploclada*) is the maternal donor of hexaploid *E. crus-galli*.

**Figure 1.**
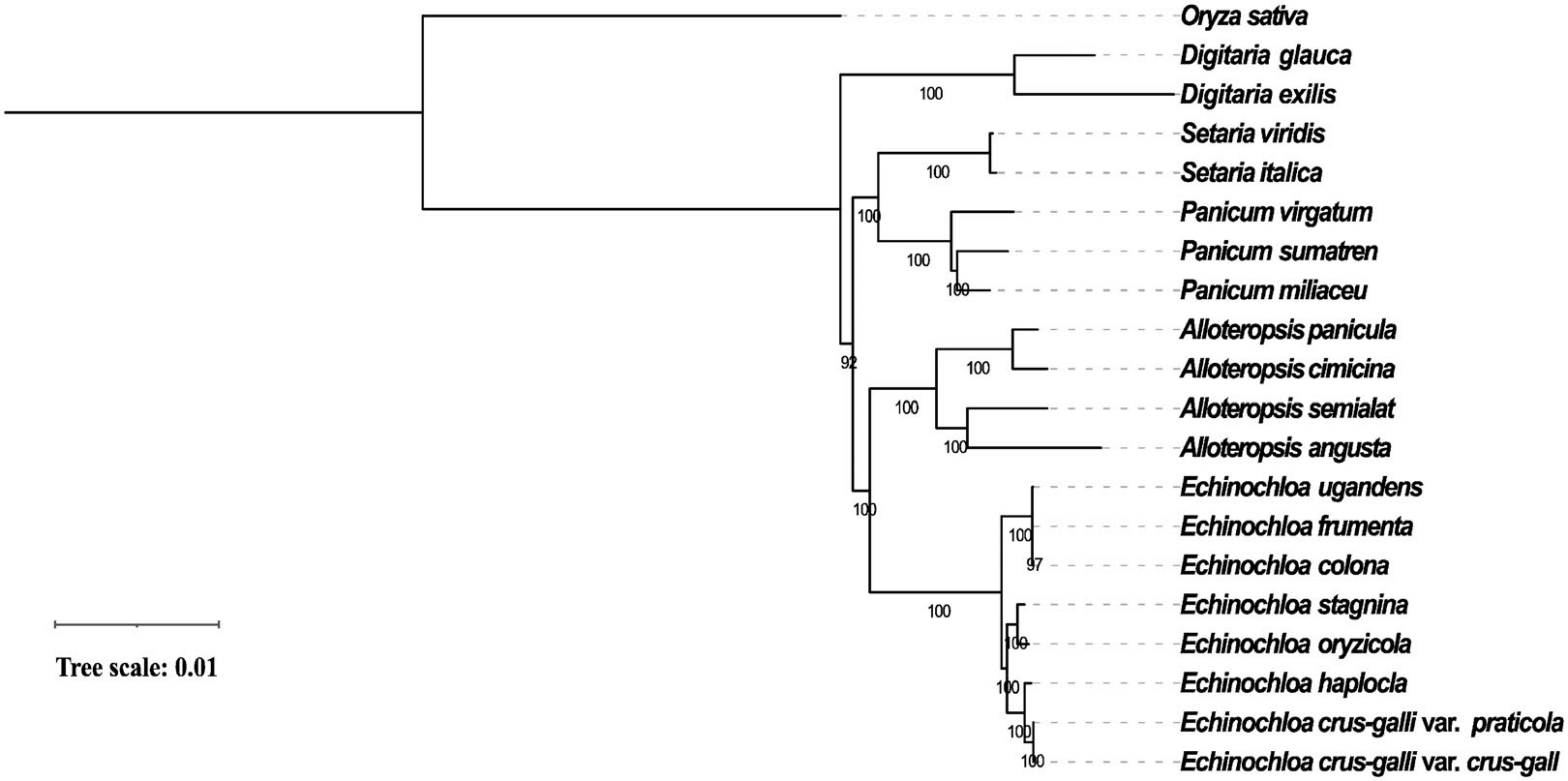
Maximum-likelihood phylogenetic tree of eight *Echinochloa* species (varieties) based on complete chloroplast genomes (*E. ugandensis*, NC_036127; *E. colona*, KT983255; *E. frumentacea*, KU242342; *E*. *oryzicola*, KJ000048; *E. stagnina*, MF563381; *E. crus-galli* var. *grus-galli*, KJ000047; *E.crus-galli* var. *praticola*, KR822686; *A. angusta*, KX752090; *A. cimicina*, MT950760; *A. paniculata*, NC_032078; *A. semialata*, MT950759.1; *D. exilis*, NC_024176; *D. glauca*, MK593550; *S. italica*, KJ001642; *S. viridis*, NC_028075; *P. miliaceum*, NC_029732; *P. sumatrense*, NC_032378; *P.virgatum*, NC_015990) with *O. sativa* as an outgroup (NC_031333). Bootstrap support value from 1000 replicates is shown on each node.

## Data Availability

The genome sequence data that support the findings of this study are openly available in GenBank of NCBI at [https://www.ncbi.nlm.nih.gov] (https://www.ncbi.nlm.nih.gov/) under the accession no. MW672445.1. The associated BioProject, SRA, and Bio-Sample numbers are PRJNA752977, SAMN20668670, and SRS9707358 respectively.
